# Case Report: A *Candida* Meningitis in an Immunocompetent Patient Detected Through the Next-Generation Sequencing

**DOI:** 10.3389/fmed.2021.656066

**Published:** 2021-10-22

**Authors:** Xiao-guang Cao, Chuang-wei Yu, Shu-sheng Zhou, Yu Huang, Chun-yan Wang

**Affiliations:** ^1^Department of Emergency Intensive Care Unit (EICU), The First Affiliated Hospital of University of Science and Technology of China (Anhui Provincial Hospital), Hefei, China; ^2^Department of Emergency Intensive Care Unit (EICU), TaiHe County People's Hospital, Fuyang, China

**Keywords:** next-generation sequencing, central nervous system infections, *Candida albicans*, fungal infections, antimicrobial therapy

## Abstract

**Background:** Fungal infections of the central nervous system (CNS) are not commonly seen clinically. Clinical diagnosis of fungal infections often depend on the pathogen culture and the clinical features. This method is time-consuming and insensitive, which can lead to misdiagnosis. The authors introduce an adult patient with fungal infections diagnosed by next-generation sequencing (NGS).

**Case:** The patient was a 60-year-old male Chinese who had both hypermyotonia of the lower extremities and fever. The auxiliary examinations such as MRI, CT, and cerebrospinal fluid (CSF) analysis showed obvious abnormalities. Because of the difficulties in diagnosis, it was hard to determine the treatment plan. The NGS detected specific sequences of *Candida albicans* in 3 days. The patient was then treated with liposomal amphotericin B and fluconazole. About 3 weeks later, the symptoms of the patient improved significantly and he was discharged from the hospital.

**Conclusion:** Compared with the routine cultural method, NGS has made a huge advancement in infection diagnosis and targeting antimicrobial therapy for CNS infection.

## Introduction

Fungal infections include *Candida* infection, cryptococcal infection, *Pneumocystis carinii* infection. (1). It is well-known that fungal infections are serious opportunistic infectious diseases, although the proportion of patients with fungal infections is not high. Moreover, based on the current epidemiological survey (2), the prevalence of fungal infections is increasing every year. There are at least 15 distinct *Candida* species that cause human disease, but >90% of the invasive disease are caused by the five most common pathogens such as *C. albicans, C. glabrata, C. tropicalis, C. parapsilosis*, and *C. krusei* (3).

Recently, invasive fungal infections (IFIs) have gained importance in public health due to the increasing number of patients with immunocompromised who are at risk for the infections caused by these opportunistic pathogens (4), especially the fungal infections of the central nervous system (CNS) (5).

Because most of these cases of fungal CNS infection are chronic and develop in patients with an altered immune response, it is difficult for clinicians to diagnose early from the clinical manifestation. The development of fungal CNS infections also mainly depends on the interplay between the health status and fungal virulence factors of the patients.

As we know, in clinical cases, the most common fungal pathogens include *Cryptococcus* spp., *Aspergillus* spp., *Histoplasma capsulatum*, and *Candida albicans*. *Candida* meningitis is the most common fungal pathogen in CNS in clinical cases (6).

Although these diseases are relatively rare, these lethal infections in the CNS give rise to significant morbidity and mortality. Meanwhile, the diagnosis of the fungal infection is often difficult and the treatment is often delayed. There have been catastrophic results because of the misdiagnosis and difficulty in the early diagnosis (5, 6). Patients with cerebrovascular disease are more likely to suffer from the fungal infection of the CNS. The incidence was related to invasive operations, broad-spectrum antibiotics abuse, and the immune system and immunodepression of the patients.

In this case, the pathogens were not found by the cerebrospinal fluid (CSF) and blood culture test. At the same time, we could not get any help from MRI and CT. The treatment of the patients is in a dilemma. Then, the next-generation sequencing (NGS) method was used to detect the pathogen and the result showed that the pathogen was *Candida*.

## Case

A 60-year-old male had a history of fever for half-month (January 1, 2019), diarrhea, 1-week history of somnolence, and extremity of hypertonia. Apart from the past medical history of chronic bronchitis and surgery of hernia, we did not get any meaningful history from his family.

When the symptoms began, he had a fever and the highest body temperature of the patient was 39°C. On the next day, he began to have incontinence and diarrhea. The patient was then sent to the Huainan Xinhua Hospital. The CT of the chest showed that he had pulmonary inflammation; the patient was given the treatment of anti-infection, but the symptoms were not alleviated. About 4 days later, the patient was transferred to the Huainan First People's Hospital and he required the serial lumbar punctures as his intracranial pressure raised persistently. The CSF test showed the abnormal results that CSF protein level and pressure were higher than usual (family members could not provide the accurate results and anti-infective agents). Meanwhile, the body temperature of the patient still fluctuated between 37.5 and 38.1°C. By using the CSF culture and virus antibodies, no pathogen was detected, so the patient continued to receive empiric treatment for viral meningitis and supportive care. Then, the patient developed the symptoms of cough and phlegm, with paroxysmal fever as high as 38.1°C. Because the symptoms of the patients did not improve, he was transferred to the Anhui Provincial Hospital. On arrival, the patient was afebrile and had a blood pressure of 127/80 mm Hg and a heart rate of 72 beats/min. The physical examination showed that he had neck stiffness and hypermyotonia. He had no focal motor or sensor deficits and his nerve examination was normal.

As a result of these data and information, the doctors considered that the patient had viral encephalitis and he was treated with gammopathy, methylprednisolone, and anti-virus therapy. Viral antibodies and β-1,3 glucanase were used to speculate on the type of pathogen again. Combined with the previous examination results of the patients in the Huainan and the current examination results, we did not get the valuable results to identify the pathogens. Therefore, the patient continued to receive empiric treatment for the viral encephalitis and supportive care, but the symptoms were not alleviated. Due to the tetanus antibody that is probable positive, the patient was transferred to the infectious department (January 13, 2019). According to the clinical symptoms, the doctors did not consider the possibility of tetanus. Then, the patient again returned to the neurology department. When the patient arrived, he showed unconsciousness and his oxygen saturation of blood decreased. For further treatment, the patient was sent to the intensive care unit (ICU) (January 15, 2019). Subsequently, the lumbar puncture was performed again, showing an open pressure of 80 mm Hg. The CSF test showed glucose of 2.48 mmol/l, protein of 0.66 g/l, karyocyte count of 10^10^~6/L, and chloride of 115.2 mmol/l (Table 1). Repetitive serology and CSF studies for the possible organisms continued to be negative. Gram staining and India ink staining did not show any organisms. CSF culture was repeated. While MRI of the brain revealed the abnormal enhancement of meningitis and hemangioma, we did not get any valuable information from MRI.

**Table 1:** Cerebrospinal fluid test.

Open Pressure		80 mmHg
Colors		Transparent
Cell Count	Karyocyte Cell	10 × 10^∧^6/L
	Red Blood Cell	0
	Mononuclear Cell	100%
	Multinucleated Cell	0%
Glucose		2.48 mmol/L
Protein		0.66 g/L
Chloride		115.2 mmol/L
Ig-G		54.6 mg/L
Ig-A		9.50 mg/L
Ig-M		1.31 mg/L

**Figure 1:**
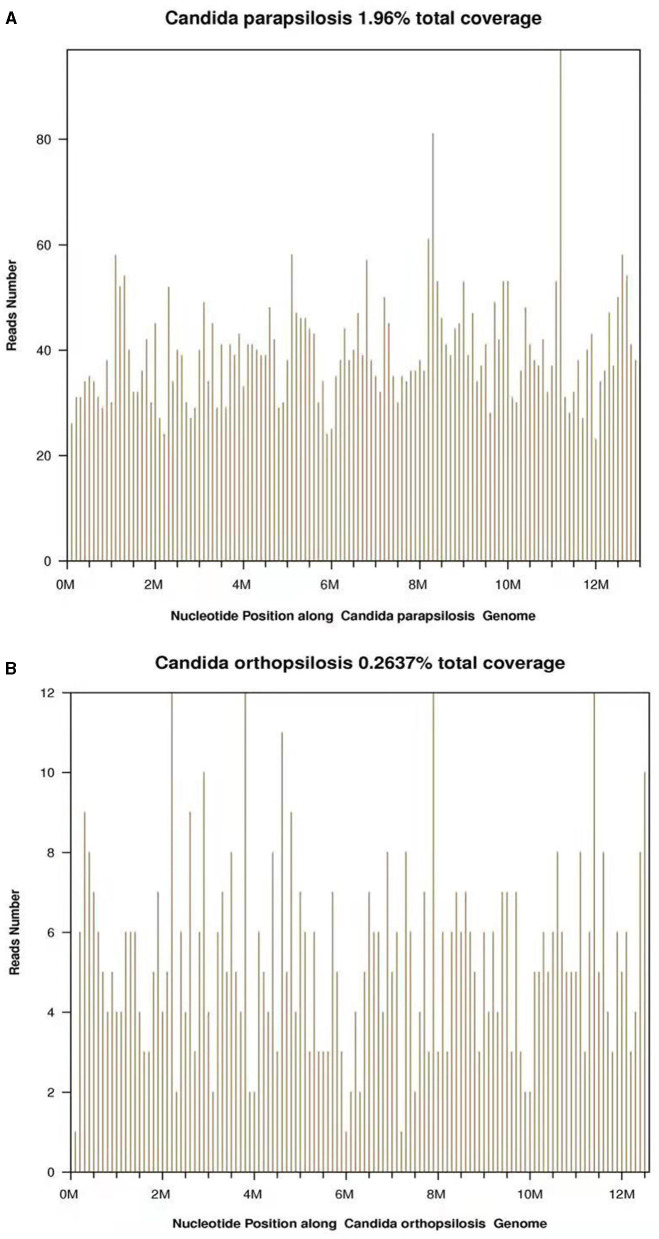
**(A)** A total of 5,212 reads were mapped to the *Candida parapsilosis* in the reference database. The coverage of the referenced *Candida parapsilosis* genome was 1.96%. **(B)** A total of 675 reads were mapped to the *Candida orthopsilosis* in the reference database. The coverage of the referenced *Candida orthopsilosis* genome was 0.2637%.

During this period, the C-reactive protein (CRP) of the patient, mental state, and body temperature gradually decreased after the antibacterial treatment. His sample was sent for pathogen detection by the NGS at the Beijing Genomics Institute (BGI), Wuhan, China. CSF sample from the patient was analyzed by using the BGI PMSeq high-throughput sequencing process for the detection of pathogenic microorganisms. DNA from the CSF sample was extracted by using the TIANamp Micro DNA Kit (Beijing, China). Then, 100 ng of extracted DNA were subjected to the library building steps including fragmentation, end repair, and sequencing adapter ligation. Then, the DNA library was sequenced on the BGISEQ-500 sequencing platform at the BGI, Wuhan, China. A total of 3,310,880 single-end reads were generated. Then, the reads were processed by using the BGI metagenome analysis platform. After filtering of the human genome sequences, the rest sequences were mapped to a metagenome database including 2,328 bacteria, 199 fungi, 4,189 viruses, and 135 parasites from the National Center for Biotechnology Information (NCBI). Among these reads, 30,451 were mapped to *Candida parapsilosis* (Figure 1A) and 2,857 were mapped to *Candida orthopsilosis* (Figure 1B), all these are fungal species. The other reads were mapped to the common environmental microbes or laboratory contaminants.

**Figure 2:**
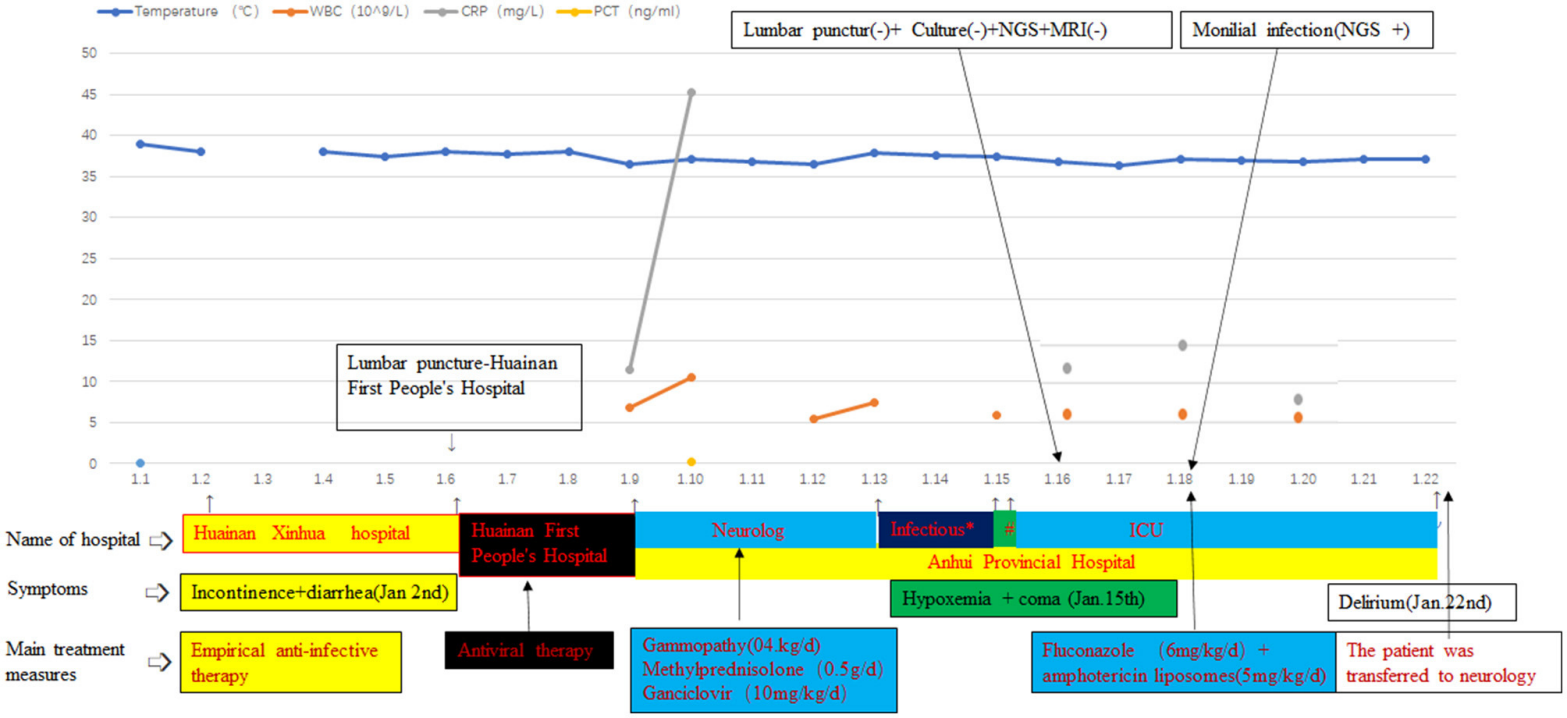
Annotation: “*” indicated the department of infection; “#” indicated that the patient was treated in the neurology department in the afternoon and he was transferred to the intensive care unit (ICU) in the evening; “–” means negative; and “+” means positive. The beginning and end of each color bar indicated the treatment measures during this period.

Due to this result, there was a debate among the doctors. Doctors were divided into two groups. One group thought that the foundation of the fungal infection was not sufficient at the current stage and the epidemiological investigation in China showed that the incidence of *Candida* infection in the CNS was low, so they thought that the possibility of contamination of the specimen cannot be ruled out. Because antithyroglobulin antibodies in the patient were positive, combined with thyroid insufficiency and MRI, they considered the possibility of Hashimoto's encephalitis, and the patient should be treated with the hormones. For these reasons, they believed that antifungal therapy was not essential. In the opinion of the other group, although there was no other evidence of the pathogens at present and some clinical symptoms of the patient improved, his muscle tension was still high. Meanwhile, there was no better detection method in our hospital. According to the Infectious Diseases Society of America (IDSA) guidelines (recommendations 28, 32, 33, and 94) (3), the change of the clinical symptoms is the most important basis for the clinicians without basis. Furthermore, the empirical treatment of adults with encephalitis is with drugs within 6 h of suspicion (7), and delay in the diagnosis and inappropriate therapy would result in permanent damage, even also death. It was an unacceptable outcome. Owing to the poor therapeutic effect and symptoms, diagnostic treatment can be initiated and the neurologists also agreed with the treatment. Then, the patient finally received the antifungal treatment with fluconazole combined with the amphotericin liposomes (the dose of fluconazole on the first day was 12, 6 mg/kg/day; amphotericin liposomes: 5 mg/kg/day) (3) and the neurologists and the family of the patient agreed with this treatment. The clinicians monitored the side effects of this treatment regimen such as hypokalemia and renal impairment. Fortunately, after 4 days of treatment, there was a significant improvement in the muscle tone without the side effects. Because of the enclosed environment of the ICU, the patient developed some mental symptoms (restlessness and delirium appeared intermittently). Because the family members are not allowed to accompany in the ICU, the patient was transferred to the neurology unit for further treatment. Doctors hope to treat delirium through the company and psychological comfort of the families of the patient (the hospitalization process is shown in Figure 2).

## Discussion

In the previous studies, the fungal infections of the CNS were rare clinical cases, but candidemia cases have increased dramatically worldwide (8, 9). Fungal infections can develop *via* hematogenous dissemination from a distant focus such as central venous catheter and lung (10). The symptoms of *Candida* meningitis are often similar to those in patients with bacterial meningitis such as fever (hyperthermia), headache, neurological symptoms, and meningeal irritation (11).

According to the results of the current research (7), a high index of suspicion is needed in any patient with risk factors such as abdominal surgery, bowel perforation, recent broad-spectrum antibiotic therapy, intravenous drug use, advanced in years, indwelling catheters, low immune function, severe burns, antineoplastic therapy, and long-term glucocorticoid usage (12, 13). The development of fungal infections largely depend on the interplay between the health status of the patients and the virulence of pathogenic fungal species. In many cases, there is no specificity in the clinical symptoms. As we know, fungal meningitis is a common complication of immunosuppression, while *Candida albicans* is the most common pathogen (14, 15). Overall, *albicans* were the most common strains followed by *Candida glabrata* in the clinical cases, accounting for about half of the total cases, but the source of candidemia was unknown in half of all the episodes (16).

In this case, the patient lacked any known risk factors, and the reason for the infection needed further study. According to the current diagnosis, fungal infection can be isolated from blood and CSF, which is the gold standard of the clinical diagnosis. However, the positive rate in the blood cultures and CSF is too low for the clinician. Even if the etiological culture is positive, it will take 3–5 days at the fastest. With respect to the timing of initiation of empirical treatment, some studies are still controversial, with some showing that mortality was lower in the patients who received the antifungal early (7, 17), while other studies showed that there was no significant difference in in-hospital mortality (18, 19).

With most of the research results, the prompt initiation of the antifungal therapy was essential for better outcomes. But in any case, timely and effective treatment with the appropriate anti-infective drugs is the most crucial factor for the best prognosis (20). Providing effective treatment for the patients is the most crying need for the patients and doctors. Meanwhile, if we can get accurate results earlier, the therapeutic efficacy will be better and fewer irrational antifungal drugs will be used. However, in most of the cases, the clinicians did not have adequate laboratory tests at the beginning; they can only judge the condition according to the guidelines, expert consensus, and clinical experience, so as to minimize the possibility of missed diagnosis, misdiagnosis, and overdiagnosis and treatment (3, 7).

The incorrect usage of the antimicrobials will cause the resistance of the pathogen, treatment difficulty, and the tensional doctor-patient relationship, especially in China (21). Fungal cultures of CSF have a bad sensitivity and this method is rarely helpful. Due to the poor sensitivity of the cultures, the diagnosis of fungal infection is very tricky (22). In addition, unlike the other infection, there is not a widely accepted molecular-, antigen-, or nucleic acid-based testing method to expedite the identification of the organism in the medical units, thus delaying the proper treatment (23). Now, diagnoses of these diseases are still tricky because there lacked a fast and reliable means of detection. Delay in the diagnosis and inappropriate therapy can result in permanent damage, even death. It was an unacceptable outcome.

Clinical doctors need another simple and effective method to isolate and detect the pathogen. In the current practice, culture-based methods are still considered as the standard approach of etiological diagnosis of the infection. However, these methods have disadvantages including long turnaround time and practically low sensitivity. Although molecular assays such as PCR-based methods have been established for the pathogen, the detection is limited to the known pathogens listed on the panel.

The NGS is a new laboratory detection technology that directly extracts the nucleic acids from all the pathogens in the samples of the patient for high-throughput sequencing; the NGS is able to detect the thousands of fragments in a laboratory test, therefore this technology can reduce the number of tests and period of detection (24). In the past few years in China, NGS is an emerging innovative technology, which has become highly sensitive in the detection of pathogens (24). NGS has been constantly improving and stepped into clinical practice, providing a powerful tool with advantages including short turnaround time, full-spectrum, and semi-quantitative analysis. In comparison to the routine cultural method, NGS has made a huge advancement in infection diagnosis. NGS had become a new and innovative technology and is gradually applied in clinical practice. In this case, the NGS results showed that pathogens were *Candida parapsilosis* and *Candida orthopsilosis*. They are the different genotypes of *Candida* (25), but only *Candida parapsilosis* is stated in this clinical guideline (3). This also proved the advantages of NGS (19).

The antimicrobial resistance profile of *Candida* in China showed that the isolates were sensitive to the liposomal amphotericin B and fluconazole. This is consistent with the IDSA guidelines (5) and this case indirectly proved the sensitivity and specificity of NGS.

The limitations included few acknowledged criteria of the sequencing result explanation which have been established (26); large-scale study comparing NGS with traditional etiological diagnosis methods which are missing, the sample size of most of the studies is small (27); this study is just a case report, thus, it still needs high-level evidence to support its clinical application; and this method is very sensitive, so strict aseptic operation is required in the detection process, else it is easy to lead to the clinical misdiagnosis (28).

## Conclusion

Next-generation sequencing technology is a new method of diagnosis. Based on the unique advantages of the NGS technology in the diagnosis of these infectious diseases, this new technology will gradually be applied in clinical practice to benefit a large number of patients.

## Data Availability Statement

The datasets presented in this study can be found in online repositories. The names of the repository/repositories and accession number(s) can be found at: Genome Sequence Archive [BioProject: PRJCA006605/CRP003428; https://bigd.big.ac.cn/gsa/browse/CRA005014].

## Author Contributions

CY conceived the idea of this article. XC and CW have made the same contributions to the article and they contributed to the preparation of the manuscript and interpreting the data of the patients. All the authors contributed to review, edit, read, and approved the final manuscript.

## Author Disclaimer

All the claims expressed in this article are solely those of the authors and they do not necessarily represent those of their affiliated organizations or those of the publisher, the editors, and the reviewers. Any product that may be evaluated in this article, or claim that may be made by its manufacturer, is not guaranteed or endorsed by the publisher.

## Conflict of Interest

The authors declare that the research was conducted in the absence of any commercial or financial relationships that could be construed as a potential conflict of interest.

## Publisher's Note

All claims expressed in this article are solely those of the authors and do not necessarily represent those of their affiliated organizations, or those of the publisher, the editors and the reviewers. Any product that may be evaluated in this article, or claim that may be made by its manufacturer, is not guaranteed or endorsed by the publisher.
